# Bevacizumab plus octreotide and metronomic capecitabine in patients with metastatic well-to-moderately differentiated neuroendocrine tumors: the xelbevoct study

**DOI:** 10.1186/1471-2407-14-184

**Published:** 2014-03-14

**Authors:** Alfredo Berruti, Nicola Fazio, Anna Ferrero, Maria Pia Brizzi, Marco Volante, Elisabetta Nobili, Lucia Tozzi, Lisa Bodei, Mirella Torta, Antonio D’Avolio, Adriano Massimiliano Priola, Nadia Birocco, Vito Amoroso, Guido Biasco, Mauro Papotti, Luigi Dogliotti

**Affiliations:** 1 Oncologia Medica, Dipartimento di Specialità Medico-Chirurgiche, Scienze Radiologiche e Sanità Pubblica, Università degli Studi di Brescia, Azienda Ospedaliera Spedali Civili Piazzale Spedali Civili 1, 25123 Brescia, Italy; 2 Istituto Europeo di Oncologia IEO, NET study group, Via Ripamonti 435, 20141 Milan, Italy; 3 Oncologia Medica, Dipartimento di Scienze Cliniche e Biologiche, Università di Torino, AOU, San Luigi, Regione Gonzole, 10, 10043 Orbassano, TO, Italy; 4 Anatomia Patologica, Dipartimento di Scienze Cliniche e Biologiche, Università di Torino, AOU, San Luigi, Regione Gonzole, 10, 10043 Orbassano, TO, Italy; 5 Dipartimento “Seràgnoli” di Scienze Ematologiche ed Oncologiche, Università di Bologna, Ospedale Sant’Orsola-Malpighi, Via G. Massarenti 9, 40138 Bologna, Italy; 6 Oncologia Medica, I.R.C.C.S. Ospedale Casa Sollievo della Sofferenza, Viale Cappuccini 3, 71013 San Giovanni Rotondo, FG, Italy; 7 Laboratorio di Farmacologia Clinica e Farmacogenetica, Dipartimento di Malattie Infettive, Università di Torino, Ospedale Amedeo di Savoia, C.so Svizzera 164, 10149 Torino, Italy; 8 Radiologia, Dipartimento di Radiologia Diagnostica e Interventistica, Università di Torino, A.O.U. San Luigi, Regione Gonzole, 10, 10043 Orbassano, TO, Italy; 9 Centro Oncologico Ematologico Subalpino COES, Azienda Ospedaliera Molinette, C.so Bramante 88, 10126 Torino, Italy

**Keywords:** Bevacizumab, Pancreatic endocrine tumor, Capecitabine, Octreotide

## Abstract

**Background:**

We assessed the activity and toxicity of the XELBEVOCT regimen in patients with metastatic well-to-moderately differentiated neuroendocrine neoplasms (WMD-NEN). Ancillary studies evaluated hypertension, proteinuria, and vascular endothelial growth factor (VEGF) polymorphisms in predicting progression-free survival (PFS) and the predictive role of serum vitamin D in progression-free survival and proteinuria onset.

**Methods:**

This prospective phase 2 study included 45 patients with WMD-NEN arising from various primary sites. The treatment regimen was octreotide long-acting release (LAR), 20 mg monthly, metronomic capecitabine, 2000 mg/daily, and intravenous bevacizumab, 5 mg/kg every 2 weeks, without interruption for 9 months. Bevacizumab was continued until disease progression.

**Results:**

Partial response was obtained in 8 patients (17.8%, 95% confidence interval [CI], 6.4%-28.2%); tumor response was more frequent in pancreatic than in non-pancreatic malignancies. The median PFS was 14.9 months; median overall survival was not attained. Biochemical and symptomatic responses were observed in 52.9% and 82.3% of cases, respectively. The treatment was well tolerated. Grade 3 toxicities included hand and foot syndrome (11.1%), proteinuria (4.4%), and renal toxicity (2.2%). Proteinuria (all grades) was correlated with longer PFS (p = 0.017). There was an inverse relationship between proteinuria and vitamin D levels. VEGF polymorphisms were not associated with patient outcome.

**Conclusion:**

The XELBEVOCT regimen is active and well tolerated in patients with metastatic WMD-NEN. Proteinuria correlated with hypovitaminosis D status and was the best predictive factor of treatment efficacy.

**Trial registration:**

Trial registration number NCT01203306.

## Background

Neuroendocrine neoplasms constitute a highly heterogeneous spectrum of tumors with a variety of biological and clinical behaviors. Site-specific classification systems generally recognize at one end of the spectrum highly aggressive, poorly differentiated neoplasms, termed neuroendocrine carcinoma grade 3 of the gastrointestinal tract and pancreas according to the new World Health Organization (WHO) classification 2010 [1], and small and large cell neuroendocrine carcinomas of the lung [2]. At the other end is a highly heterogeneous group of well-to-moderately differentiated neuroendocrine neoplasms (WMD-NEN) comprising low-grade (G1) and intermediate grade (G2) neuroendocrine tumors of the gastrointestinal tract and pancreas [1], typical and atypical carcinoids of the lung and thymus [2], along with other cancers such as medullary thyroid carcinoma and pheochromocytoma/paraganglioma. These neoplasms are relatively rare and, despite their relatively indolent course, are frequently diagnosed in advanced stage [3,4]. Because of their limited aggressiveness, WMD-NEN are notoriously resistant to standard chemotherapy and are usually addressed with biological target therapies [5].

For decades the standard therapy for both functioning and non-functioning WMD-NEN has been with somatostatin analogues [6,7]. More recently, target drugs, such as sunitinib and everolimus, have been shown to be efficacious in the management of pancreatic WMD-NEN [8,9]. One of the antiangiogenesis-targeted drugs that can be added to other types of chemotherapy is bevacizumab, a monoclonal antibody that blocks vascular endothelial growth factor (VEGF) by binding to its receptor. In a randomized phase 2 trial, the combination of bevacizumab and octreotide had a greater efficacy than interferon α2 and octreotide [10]. Common toxicities of bevacizumab are hypertension and proteinuria; both these adverse events may lead to premature interruption of drug administration. Hypertension, however, has been associated with drug efficacy in breast cancer and colon cancer patients [11,12], and proteinuria has been suggested as a potential marker of drug efficacy [13]. In addition, VEGF polymorphisms have been found to be predictive of hypertension and associated with time to progression in advanced breast cancer treated with bevacizumab [11].

Hypovitaminosis D is highly prevalent in cancer patients. It has been reported to increase the risk of cardiovascular diseases and mortality in such patients [14] and to promote proteinuria in patients with type 2 diabetes [15]. To our knowledge, the association of hypovitaminosis D and bevacizumab-induced proteinuria has never been explored to date nor has the prognostic role of hypovitaminosis D been tested in patients with neuroendocrine tumors.

Metronomic administration of chemotherapy inhibits angiogenesis and vasculogenesis by continuously exposing the more slowly proliferating tumor endothelial cells to the damaging action of the cytotoxic therapy [16]. These requisites make metronomic chemotherapy a suitable approach in the management of WMD-NEN. In our previous phase 2 trial, we observed that the combination of octreotide long-acting release (LAR) therapy 20 mg every 4 weeks plus 5-fluorouracil protracted continuous infusion led to a disease control rate >90% (response + stabilization) as first-line treatment in the management of locally advanced or metastatic WMD-NEN [17]. In contrast, the response rate obtained with octreotide LAR alone is generally <5% [6] and the time to progression reported in the PROMID trial with octreotide LAR 30 mg every 4 weeks was 14 months [18].

There is a strong rationale for combining metronomic chemotherapy with antiangiogenic drugs [19]. Prior preclinical metronomic chemotherapy studies have shown that the combination of antiangiogenic drugs with metronomic chemotherapy can increase antitumor efficacy compared to either agent alone [19,20]. Capecitabine is an orally administered third-generation fluoropyrimidine carbamate that mimics the tolerability and activity of protracted intravenous 5-FU infusion [21].

The XELBEVOCT trial, a single-arm multicenter Italian phase 2 study, was designed to test the activity of the association of metronomic capecitabine, bevacizumab, and octreotide in the treatment of advanced/metastatic WMD-NEN. The primary study aim was the assessment of activity; the secondary aims were to evaluate toxicity, time to progression, overall survival, and the relationship between common toxicities associated with bevacizumab (hypertension and proteinuria) and drug efficacy. In predefined ancillary studies we also explored the role of circulating VEGF, VEGF polymorphisms, vitamin D status, and vitamin D metabolism-related polymorphism in predicting toxicities and treatment efficacy.

## Methods

### Patients

Eligibility criteria for inclusion in this trial were: histologically or cytologically proven diagnosis of WMD-NEN; metastatic or inoperable disease not previously treated with chemotherapy; age ≥18 years; progressive disease as documented by Response Evaluation Criteria in Solid Tumors (RECIST); Eastern Cooperative Oncology Group (ECOG) performance status 0–2; life expectancy of at least 12 weeks; measurable and/or evaluable lesions according to RECIST; adequate bone marrow reserve (neutrophils ≥1500/mm^3^ and platelets ≥80,000/mm^3^); hemoglobin ≥9.0 g/dl; total bilirubin ≤1.5 times the upper limit of normal; prothrombin time-international normalized ratio/partial thromboplastin time (PT-INR/PTT) <1.5 times the upper limit of normal; serum creatinine ≤1.5 times the upper limit of normal; potential; ability to comply with the protocol procedures. Previous treatments with a somatostatin analogue and/or radionuclide therapy were permitted.

Exclusion criteria were: serious non-healing wounds or ulcers; evidence of bleeding diathesis or coagulopathy; uncontrolled hypertension; clinically significant cardiovascular diseases or cerebrovascular accidents; daily treatment with anticoagulants and high-dose aspirin (>325 mg/day); use of an investigational drug within 30 days prior to enrollment; known allergy to any of the components of the study medications; other malignancies diagnosed within the last 5 years, except basal cell carcinoma or cervical cancer *in situ*; major surgical procedures 28 days prior to study treatment start; and pregnant or lactating women. This study was first approved by the Ethic Committee of the Azienda Ospedaliera San Luigi, Orbassano, Italy. The study was approved by the local ethics committee of each participating study center. Written informed consent was obtained from all patients before starting treatment.

### Histology and biochemical analyses

The histological diagnoses performed at each center were centrally reviewed by two experienced pathologists (MV, MP). Neuroendocrine phenotype was confirmed by means of chromogranin A (clone LK2H10, diluted 1:800; NeoMarkers, Fremont, CA, USA) and synaptophysin (clone Sy38, diluted 1:100, DakoCytomation, Glostrup, Denmark) immunostaining. WMD-NEN, irrespective of the primary tumor site, were characterized by an organoid growth pattern and a mitotic rate <20 in 2 mm^2^ and the absence of extensive necrosis. In addition, in all cases in which residual tumor tissue was available, immunohistochemical analyses of Ki-67 (clone MIB-1, diluted 1:150, Dako) and somatostatin receptor type 2A (polyclonal, code SS-800, diluted 1:3000, BioTrend, Cologne, Germany) were performed. Well-differentiated tumors were defined as those with a Ki-67 proliferation index ≤2% and/or a mitotic rate <2; moderately differentiated tumors were defined as those with a Ki-67 proliferation index between 3% and 20% and/or a mitotic rate between 2 and 20 [1,22]. Plasma samples were obtained from patients who consented to having optional blood draws for analysis of VEGF (kit) and VEGF polymorphisms, circulating vitamin D (25-OH colecalcipherol) and vitamin D polymorphism. Vitamin D polymorphism (rs4646536) and VEGF polymorphisms (rs699947 and rs1570360) were assessed using the ABI TaqMan allelic discrimination kit by real-time PCR with standard methodology.

### Treatment schedule

Octreotide LAR therapy was administered at a dose of 20 mg every 4 weeks. Capecitabine was administered orally on a metronomic schedule at a dose of 1000 mg twice daily without interruption for a maximum of 9 months. Treatment was discontinued earlier in case of unacceptable toxicity, patient’s withdrawal of consent or evidence of disease progression. Continuation of capecitabine therapy beyond 9 months was permitted according to the investigator’s discretion. Bevacizumab was given intravenously at a dose of 5 mg/kg every 2 weeks. Bevacizumab treatment was planned to be continued for 9 months in association with capecitabine. Maintenance bevacizumab treatment until progression or unacceptable toxicity was prescribed in patients at the individual investigator’s discretion.

Bevacizumab doses were temporarily discontinued if proteinuria was >2 g per 24 hours or other grade 3 or 4 toxicities developed. Bevacizumab was permanently withdrawn if grade 4 hypertension or grade 4 thrombosis occurred.

The capecitabine dose was modified as follows: delayed for 1 week if granulocyte counts were <1.5 × 10^9^/L and/or platelet counts were <100 × 10^9^/L; interrupted until improvement to grade 0–1 in the event of hand and foot syndrome or severe palmar-plantar erythema with blistering and desquamation; discontinued for 1 week in case of persistent diarrhea, otherwise antidiarrheal agents were prescribed for grade 1 or 2 diarrhea; stopped for 1 week in case of grade 2 mucositis; interrupted until recovery in the event of grade 3 or 4 mucositis, then restarted at a 25% reduction in dosage.

### Treatment evaluation

Pretreatment and on-study evaluations included: history; physical examination; laboratory tests; and analysis of tumor markers (chromogranin A), serum VEGF, VEGF polymorphism, vitamin D, vitamin D polymorphism. Tumor measurements were made by computed tomography (CT) scans or magnetic resonance imaging (MRI) at baseline and every 12 weeks. All radiographic images were reviewed centrally by an independent radiologist (AMP).

Activity was assessed according to RECIST version 1.0 (CT or MRI) at baseline and every 3 months.

The duration of progression-free survival and overall survival was measured starting from the date of study enrollment. Biochemical response was evaluated in patients with elevated markers at baseline. Response was defined as a >30% reduction in tumor marker levels or normalization of the elevated tumor marker. Safety was assessed according to Common Toxicity Criteria, version 3.0.

### Statistical analyses

A two-stage Simon design allowing for early stopping was used [23]. The main end point of the study was the best change in tumor size at any time point during therapy. In keeping with a classical two-stage optimal design, a proportion of responses <10% was defined as unacceptable and a response rate > 30% was considered acceptable.

With a two-sided alpha error of 0.05 and a power of 0.90 to detect a true response probability of 30%, 18 patients were entered in the first stage; if 3 or more patients responded, the trial entered the second stage and another 18 patients were recruited. The upper limit of second-stage rejection was 7 responses observed out of 36 patients enrolled. In order to limit the heterogeneity of the trial with respect to the sample size, the primary analysis was performed only in patients with gastroenteropancreatic tumors or tumors of unknown primary origin. Other tumors were recruited for analysis but analyzed separately. The distribution of PFS and OS was estimated using the Kaplan-Meier method. The median PFS and OS in each stratum was calculated with 95% confidence intervals.

## Results

### Patient population

The study opened for enrollment in November 2006 and closed to accrual in April 2009 when 45 consecutive patients were enrolled at five participating centers. The primary sites of malignancy were: the pancreas (n = 19); the intestinal tract (n = 13); the lungs (n = 8); unknown (n = 4); and a paraganglioma (n = 1). Table 1 reports patient demographics and characteristics. None of the patients had received any type of chemotherapy, 19/45 patients (42.2%) had been previously treated with somatostatin analogues, and 23/45 patients (51.1%) with radionuclide therapy before entering the study.

**Table 1:** Patient and clinical characteristics (no. of patients (%))

	**All (N=45)**	**Pancreatic (N=19)**	**Gastrointestinal intestinal and unknown origin (N=17)**	**Others lung and paraganglioma (N=9)**
**Sex**				
Male	32 (71.1)	15 (78.9)	9 (52.9)	8 (88.9)
Female	13 (28.9)	4 (21.1)	8 (47.1)	1 (11.1)
**Age (yrs)**				
Median (range)	52 (28-73)			
**ECOG performance status**				
0	36 (80.0)	13 (68.4)	17 (100)	6 (66.7)
1	6 (13.3)	3 (15.8)	--	3 (33.3)
2	3 (6.7)	3 (15.8)		
**Ki67 index**				
≤2	10 (25.6)	2 (11.8)	5 (33.3)	3 (42.8)
3-20	29 (74.4)	15 (88.2)	15 (66.6)	4 (57.2)
Missing	6	2	2	2
**Octreoscan**				
Positive	43 (95.6)	18 (94.7)	16 (94.1)	9 (100)
Negative	2 (4.4)	1 (5.3)	1 (5.9)	
**Circulating chromogranin A**				
Normal	8	3 (17.6)	4 (23.5)	1 (12.5)
Elevated	34	14 (82.4)	13 (76.5)	7 (87.5)
Missing	3	2	--	1
**Sites of metastatic disease**				
Liver	41 (91.1)	18 (94.7)	16 (94.1)	7 (77.8)
Lymph nodes	17 (37.8)	6 (31.4)	8 (47.1)	3 (33.3)
Lung	5 (11.1)	0 (0)	--	4 (44.4)
Bone	2 (4.4)	2 (10.5)	1 (5.9)	--
**Syndrome**	17 (37.8)	5 (26.3)	11 (64.7)	1 (11.1)
Diarrhea	10 (22.2)	3 (15.8)	6 (35.3)	1 (11.1)
Hot flashes	7 (15.6)	--	6 (35.3)	1 (11.1)
Rash	7 (15.6)	--	6 (35.3)	1 (11.1)
Hypoglycemia	1 (2.2)	1 (5.3)	--	--
Cushing’s syndrome	1 (2.2)	1 (5.3)	--	--
**Previous treatments**				
Surgery	34 (75.6)	13 (68.4)	14 (82.3)	7 (77.8)
Radiotherapy	2 (4.4)	2 (10.5)	--	--
Radionuclide therapy with somatostatin analogues	23 (51.1)	10 (43.5)	8 (34.8)	5 (21.7)

Chromogranin A serum levels were measured in 42 patients, 34 of which (80.9%) had supranormal values. The proportion of elevated CgA levels was unevenly distributed across the 3 patient subgroups.

### Clinical activity

**Figure 1:**
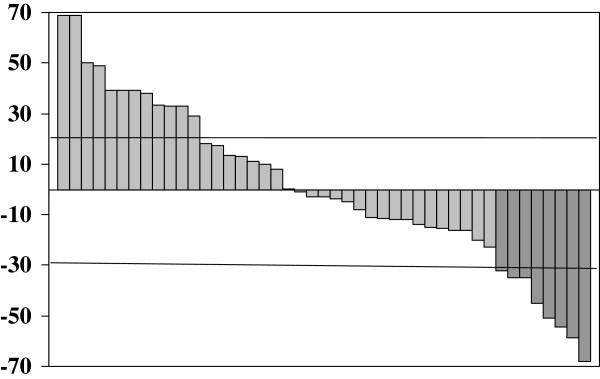
Waterfall diagram of changes in tumor size in all patients.

Forty-four out of the 45 consecutively enrolled patients could be assessed for response; the remaining patient withdrew consent to continue the experimental treatment after the first cycle and so could not be assessed. According to the intent-to-treat analysis, a partial response was observed in 8/45 patients (17.8%; 95% CI, 6.4%-28.2%), stable disease in 29 (64.4%), and disease progression in 8 (17.8%) (Figure 1). The response rate was 22.2% among the 8/36 patients with gastroenteropancreatic tumors or with tumors of unknown primary origin. When the patients were grouped according to the primary cancer site, a disease response was more frequently observed in those with pancreatic cancer (5/19, 26.3%) than in those with non-pancreatic cancer (3/26, 11.5%).

Biochemical response was evaluable in 34 patients: overall, 18/34 (52.9%) showed a CgA reduction >30% from baseline; specifically, 6/12 (50.0%) of those with pancreatic cancer, 7/14 (50.0%) with intestinal carcinoids or cancer of unknown primary origin, and 5/8 (62.5%) with lung or paraganglioma showed a biochemical response, whereas 14/17 patients (82.3%) with a specific syndrome obtained a symptomatic response.

### Progression-free survival and survival

At the last follow-up evaluation in June 2011, 35 (77.8%) patients showed disease progression and 14 (31.1%) had died. The median follow-up duration was 38 months. The median progression-free survival was 14.9 months (95% CI, 2.4-27.5) for all patients, 14.3 months (95% CI, 3.5-25.0) for those with pancreatic cancer, 14.3 months (95% CI, 0.0-38.6) for those with intestinal tumor or unknown primary cancer site, and 18.6 months (95% CI, 7.9-29.2) for those with lung (+ paraganglioma) cancer. When the patients were grouped according to the response obtained, the progression-free survival was 14.9 (95% CI, 2.4-38.6) in those who attained a partial response or stable disease and 11.4 (95% CI, 0.0-25) in those with disease progression. Median overall survival was not attained in patients with pancreatic cancer or those with intestinal or unknown primary cancer, whereas in those with lung neuroendocrine tumors or paraganglioma the median overall survival was 38 months (95% CI, 35.0-40.1).

### Treatment administered and adverse events

Capecitabine treatment was administered for a median duration of 9 months (range, 1–11). Of the 45 patients in whom treatment was initiated, 24 (53.3%) completed the scheduled 9-month treatment and 21 interrupted earlier due to: progressive disease (n = 11); withdrawal of consent (n = 5); hand and foot syndrome (n = 2); asthenia (n = 1); venous thrombosis (n = 1); renal impairment (n = 1); and hepatic chemoembolization (n = 1).

Treatment with metronomic capecitabine and bevacizumab and concomitant octreotide LAR was generally well tolerated. Most adverse events were mild to moderate in severity (Table 2). The most common adverse events considered to be related to the study drugs were nausea/vomiting, diarrhea, hand and foot syndrome, hypertension, proteinuria, and asthenia. Grade 3 toxicities included: hand and foot syndrome (n = 5,11.1%); proteinuria (n = 2, 4.4%); and renal toxicity (n = 1, 2.2%).

**Table 2:** Toxicity

	**Grade**			
	**0**	**1**	**2**	**3**
**No. of patients (%)**				
**Hematologic**				
Neutropenia	40 (88.9)	3 (6.7)	2 (4.4)	
Anemia	35 (77.8)	5 (11.1)	5 (11.1)	
**Non hematologic**				
Nausea/vomiting	29 (64.4)	13 (28.9)	3 (6.7)	
Diarrhea	23 (51.1)	17 (37.8)	5 (11.1)	
Stomatitis	40 (88.9)	4 (8.9)	1 (2.2)	
Hand and foot syndrome	17 (37.8)	12 (26.7)	11 (24.4)	5 (11.1)
Onycholysis	43 (95.6)	2 (4.4)		
Hypertension	27 (60.0)	16 (35.6)	2 (4.4)	
Proteinuria	20 (44.4)	13 (28.9)	7 (15.6)	2 (4.4)
Hemorrhage	42 (93.7)	3 (6.7)		
Thrombosis	43 (95.6)	2 (4.4)		
Cardiac	44 (97.8)	1 (2.2)		
Fever	34 (75.5)	11 (24.4)		
Asthenia	27 (60.0)	15 (33.3)	3 (6.7)	

Octreotide administration was never delayed nor reduced. Capecitabine administration was delayed for at least 1 week in 12 patients (26.7%) and for 2 weeks in 16 patients (35.6%). The most frequent reasons for the delayed course were hand and foot syndrome (n = 13), neutropenia (n = 4), and diarrhea (n = 4). The capecitabine dose was reduced by 25% in 12 patients (26.7%), with neutropenia (n = 3) and hand and foot syndrome (n = 5) being the most frequent causes. Bevacizumab was never reduced nor delayed; it was early interrupted in 2 patients because of renal impairment and deep venous thrombosis, respectively. Of the 24 patients who completed the 9-month treatment with bevacizumab, 16 received bevacizumab maintenance and 8 did not for the following reasons: deep venous thrombosis (n = 2); proteinuria (n = 1); rectal bleeding (n = 1); and investigator decision (n = 4).

### Toxicity as a predictive factor of treatment efficacy

**Figure 2:**
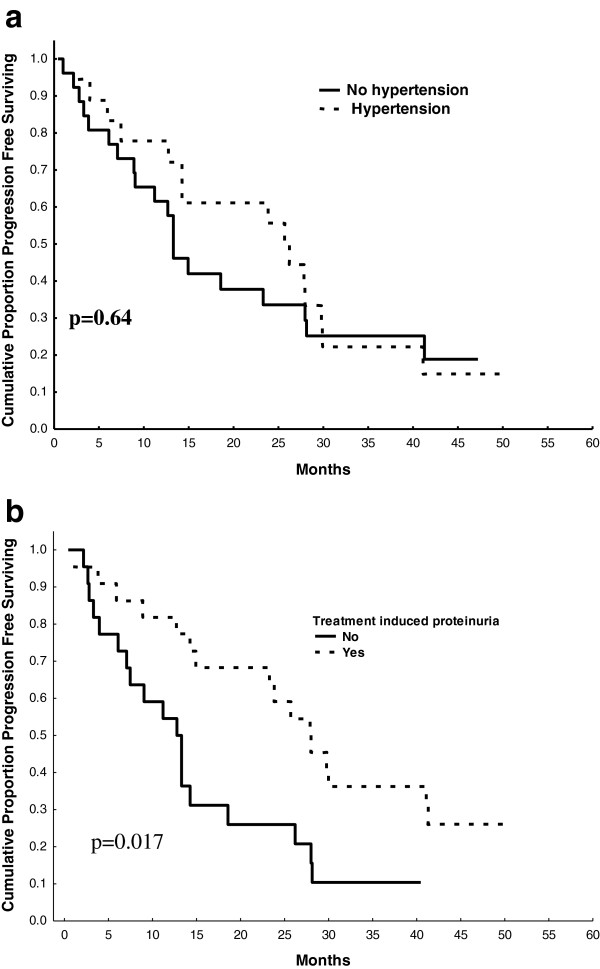
Effect of hypertension (a) and proteinuria (b) on time to progression.

Hypertension was not associated with progression-free survival (p = 0.64) (Figure 2a); however, patients who experienced proteinuria had a significant gain in progression-free survival (p = 0.017) (Figure 2b) that was maintained after adjusting for bevacizumab treatment duration (hazard ratio [HR] 0.44; 95% CI, 0.21-0.91; p = 0.028). The correlation between the occurrence of hand and foot syndrome and progression-free survival just failed to attain statistical significance after adjusting for capecitabine treatment duration (HR 0.56; 95% CI, 0.27-1.17; p = 0.13).

### VEGF and VEGF polymorphisms as predictors of bevacizumab toxicity and efficacy

Serum VEGF levels at baseline were available in 36 patients. Baseline VEGF values, dichotomized at the median value, failed to be associated with progression-free survival (HR 1.31; 95% CI, 0.61-2.79).

VEGF polymorphism was analyzed in blood samples from 28 patients. There was a non significant association between rs699947 polymorphism and hypertension: hypertension was present in7/10 patients (70.0%) with CC genotype and in 7/18 patients (38.9%) with CA/AA genotype (p = 0.11). No association was found between rs699947 polymorphism and proteinuria. No association between rs1570360 polymorphism and either hypertension or proteinuria was observed. Rs699947 and rs1570360 polymorphisms both failed to be associated with progression-free survival (data not shown).

### Serum vitamin D and vitamin D polymorphisms as predictors of treatment efficacy and toxicity

**Figure 3:**
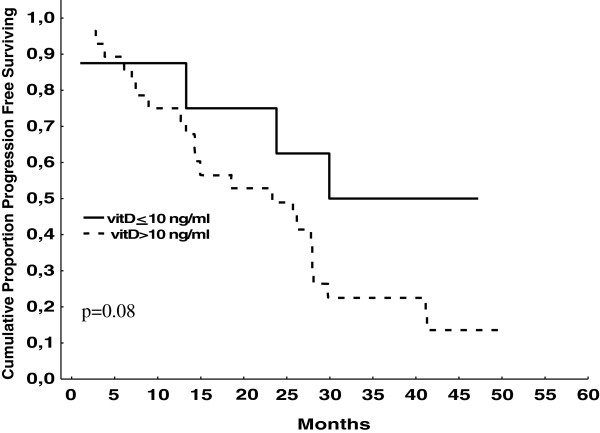
Influence of hypovitaminosis D on time to progression.

Serum vitamin D baseline values were available for 36 patients and circulating vitamin D polymorphism for 28 patients. Vitamin D status failed to be associated with hypertension; however, a significant association was found between proteinuria and the severity of hypovitaminosis D status: proteinuria was present in 6/8 patients (75.0%) with serum vitamin D <10 ng/ml, in 10/14 (71.4%) with serum vitamin D between 10 and 20 ng/ml, and in 5/14 (35.7%) with serum vitamin D >20 ng/ml (p for trend 0.049). Vitamin D deficiency (<10 ng/ml) was associated with longer PFS and approached statistical significance (Figure 3). Vitamin D polymorphism failed to be associated with hypertension, proteinuria, and PFS (data not shown).

## Discussion

In this study, the combination of octreotide LAR plus metronomic capecitabine and bevacizumab was active in the management of patients with metastatic WMD-NEN, the treatment was well tolerated, and the majority of the patients completed the treatment plan. As expected, response was better in patients with pancreatic cancer than in those with non-pancreatic cancer, and as recently recognized as the best end point of treatment efficacy in WMD-NEN [22], progression-free survival was similar in both subgroups, suggesting that the drug combination was effective in all subgroups. The similar biochemical response observed in all subgroups further supports this statement. The absence of randomization precluded determining whether the addition of bevacizumab was synergistic with capecitabine or not.

The response rate obtained in this study was similar to that observed in our previous trial with octreotide LAR plus continuous 5-fluorouracil infusion [17], but the progression-free survival was shorter. The difference between the two populations and in the inclusion criteria limits drawing comparison between the two trials. One weakness of the current trial is the preplanned stop of capecitabine and bevacizumab at the ninth month. It is unclear whether that could have influenced the clinical results, since a significant proportion of patients received bevacizumab until disease progression. Another critical point is that the efficacious dose rate in the metronomic schedule of capecitabine has not yet been well defined. From our trial it seems that toxicity is dose-dependent: administration of 2000 mg/day was associated with the occurrence of G3 hand and foot syndrome in 11% of patients, at variance with no G3 with 1500 mg/day used in another study [20].

Hypertension, a common side effect of bevacizumab linked to VEGF inhibition, is associated with a greater efficacy of this monoclonal antibody [11,12], and VEGF polymorphisms, which are predictive of bevacizumab-induced hypertension, are also correlated with the drug’s efficacy [11]. In the present study, neither hypertension nor VEGF polymorphisms were associated with progression-free survival; however, proteinuria, a consequence of glomerular injury due to direct targeting of VEGF [24], was predictive for longer progression-free survival. To our knowledge, proteinuria as a predictive factor of bevacizumab efficacy has been suggested only in a case report to date [13] but was never demonstrated in a prospective study.

Data from studies in experimental diabetic nephropathy indicate that vitamin D insufficiency may be involved in the pathogenesis of albuminuria [15]. In the general population, an inverse association was found between the level of vitamin D and the prevalence of albuminuria [25]. These observations encouraged us to explore whether vitamin D status might be associated with the onset of bevacizumab-induced proteinuria. We found an inverse relationship between serum vitamin D levels and proteinuria in the present series. Although association does not mean causality, this finding suggests that bevacizumab-induced proteinuria can be effectively treated with adequate vitamin D supplementation. Strong support for this hypothesis comes from a multicenter prospective randomized clinical trial which showed that paricalcitol administered to patients with diabetic nephropathy can lower albuminuria [26]. While proteinuria was found to be a marker of bevacizumab efficacy, vitamin D supplementation may impair the drug’s efficacy. No conclusions can be drawn from this small patient sample; nonetheless, hypovitaminosis D status was associated with a gain in progression-free survival (approaching statistical significance), and this contrasts with previously published data showing a negative prognostic role of hypovitaminosis D [26].

Circulating VEGF levels were found to be prognostic in patients with advanced WMD-NEN not previously treated with angiogenesis inhibitors [26]; however, in these patients receiving a bevacizumab-containing regimen, the marker failed to be associated with progression-free survival.

## Conclusion

In conclusion, XELBEVOCT is an active and well-tolerated regimen in the management of metastatic WMD-NEN. Proteinuria correlated with hypovitaminosis D status and was the best predictive factor of treatment efficacy. The main limitations of this study are its explorative nature and small population sample size. While the XELBEVOCT scheme cannot be applied in clinical routine, the regimen’s activity and efficacy compare favorably with those of currently available target agents such as sunitinib [8] and everolimus [9], radionuclide therapy [27] and cytotoxic therapy [4], particularly with respect to progression-free survival. The cost of this regimen is similar to that of other molecular target agents currently used in the management of neuroendocrine tumors. In our opinion, the XELBEVOCT regimen merits further testing in prospective phase 3 trials.

## Competing interest

The authors declare no competing interest.

In particular none of the authors the past five years received reimbursements, fees, funding, or salary from an organization that may in any way gain or lose financially from the publication of this manuscript, either now or in the future.

None of the authors hold any stocks or shares in an organization that may in any way gain or lose financially from the publication of this manuscript, either now or in the future. None of the authors hold or are currently applying for any patents relating to the content of the manuscript; have you received reimbursements, fees, funding, or salary from an organization that holds or has applied for patents relating to the content of the manuscript. None of the authors have any other financial competing interests, nor non-financial competing interests.

There are no non-financial competing interests (political, personal, religious, ideological, academic, intellectual, commercial or any other) to declare in relation to this manuscript.

## Authors’ contributions

AB drafted the manuscript; MPB and AF acquired the data and revising manuscript critically; NF and LB assisted in data acquisition; MV and MP performed the histological diagnoses; AMP performed the extramural radiologic review. AD and MT collected the biological data; NB, LT, EN, and GB followed the patients and collected the data. LD contributed to trial conception and design. All authors have given their approval of the final version of the manuscript.

## Pre-publication history

The pre-publication history for this paper can be accessed here:

http://www.biomedcentral.com/1471-2407/14/184/prepub

## References

[B1] Bosman FT Carneiro F Hruban RH Theise ND Tumors of the Digestive System. World Health Organization Classification of Tumours. Pathology and Genetics 2010 Lyon: IARC Press

[B2] Travis WD Brambilla E Muller-Hermelink HK Harris CC Tumours of the Lung, Pleura, Thymus and Heart. World Health Organization Classification Tumours. Pathology and Genetics 2004 2004 Lyon: IARC Press

[B3] Modlin IM Oberg K Chung DC Jensen RT de Herder WW Thakker RV de Herder WW Thakker RV Caplin M Delle Fave G Kaltsas GA Krenning EP Moss SF Nilsson O Rindi G Salazar R Ruszniewski P Sundin A Gastroenteropancreatic neuroendocrine tumours Lancet Oncol 2008 9 1 61 72 10.1016/S1470-2045(07)70410-2 18177818

[B4] Yao JC Hassan M Phan A Dagohoy C Leary C Mares JE Abdalla EK Fleming JB Vauthey JN Rashid A Evans DB One hundred years after “carcinoid”: epidemiology of and prognostic factors for neuroendocrine tumors in 35,825 cases in the United States J Clin Oncol 2008 18 3063 3072 18565894 10.1200/JCO.2007.15.4377

[B5] Volante M Righi L Berruti A Rindi G Papotti M The pathological diagnosis of neuroendocrine tumors: common questions and tentative answers Virchows Arch 2011 458 4 393 402 10.1007/s00428-011-1060-7 21344263

[B6] Fazio N Cinieri S Lorizzo K Squadroni M Orlando L Spada F Macello E Bodei L Paganelli G Delle Fave G De Braud F Biological targeted therapies in patients with advanced enteropancreatic neuroendocrine carcinomas Cancer Treat Rev 2010 36 Suppl 3 S87 S94 21129617 10.1016/S0305-7372(10)70026-8

[B7] Modlin IM Pavel M Kidd M Gustafsson BI Review article: somatostatin analogues in the treatment of gastroenteropancreatic neuroendocrine (carcinoid) tumours Aliment Pharmacol Ther 2010 15;31 2 169 188 10.1111/j.1365-2036.2009.04174.x 19845567

[B8] Raymond E Dahan L Raoul JL Bang YJ Borbath I Lombard-Bohas C Metrakos P Smith D Vinik A Chen JS Hörsch D Hammel P Wiedenmann B Van Cutsem E Patyna S Lu DR Blanckmeister C Chao R Ruszniewski P Sunitinib malate for the treatment of pancreatic neuroendocrine tumors N Engl J Med 2011 10;364 6 501 513 10.1056/NEJMoa1003825 21306237

[B9] Yao JC Shah MH Ito T Bohas CL Wolin EM Van Cutsem E Hobday TJ Okusaka T Capdevila J de Vries EG Tomassetti P Pavel ME Hoosen S Haas T Lincy J Lebwohl D Öberg K RAD001 in Advanced Neuroendocrine Tumors Everolimus for advanced pancreatic neuroendocrine tumors N Engl J Med 2011 10;364 6 514 523 10.1056/NEJMoa1009290 PMC4208619 21306238

[B10] Yao JC Phan A Hoff PM Chen HX Charnsangavej C Yeung SC Ajani JA Targeting vascular endothelial growth factor in advanced carcinoid tumor: a random assignment phase II study of depot octreotide with bevacizumab and pegylated interferon alpha-2b J Clin Oncol 2008 10;26 8 1316 1323 10.1200/JCO.2007.13.6374 18323556

[B11] Schneider BP Wang M Radovich M Sledge GW Badve S Thor A Flockhart DA Hancock B Davidson N Gralow J Dickler M Perez EA Cobleigh M Shenkier T Edgerton S Miller KD ECOG 2100 Association of vascular endothelial growth factor and vascular endothelial growth factor receptor-2 genetic polymorphisms with outcome in a trial of paclitaxel compared with paclitaxel plus bevacizumab in advanced breast cancer: ECOG 2100 J Clin Oncol 2008 28 4672 4678 18824714 10.1200/JCO.2008.16.1612 PMC2653128

[B12] Scartozzi M Galizia E Chiorrini S Giampieri R Berardi R Pierantoni C Cascinu S Arterial hypertension correlates with clinical outcome in colorectal cancer patients treated with first-line bevacizumab Ann Oncol 2009 20 2 227 230 18842611 10.1093/annonc/mdn637

[B13] Karachaliou N Saloustros E Vamvakas L Mavroudis D Georgoulias V Proteinuria and favourable clinical response in a patient receiving paclitaxel + bevacizumab for metastatic breast cancer Ann Oncol 2010 21 8 1729 1730 10.1093/annonc/mdq328 20601370

[B14] Holick MF Vitamin D deficiency N Engl J Med 2007 19;357 3 266 281 10.1056/NEJMra070553 17634462

[B15] Agarwal R Vitamin D, proteinuria, diabetic nephropathy, and progression of CKD Clin J Am Soc Nephrol 2009 4 9 1523 1528 10.2215/CJN.02010309 19478099

[B16] Kerbel RS Kamen BA The anti-angiogenic basis of metronomic chemotherapy Nat Rev Cancer 2004 4 423 436 10.1038/nrc1369 15170445

[B17] Brizzi MP Berruti A Ferrero A Milanesi E Volante M Castiglione F Birocco N Bombaci S Perroni D Ferretti B Alabiso O Ciuffreda L Bertetto O Papotti M Dogliotti L Continuous 5-fluorouracil infusion plus long acting octreotide in advanced well-differentiated neuroendocrine carcinomas. A phase II trial of the Piemonte Oncology Network BMC Cancer 2009 9 388 395 10.1186/1471-2407-9-388 19886987 PMC2776604

[B18] Rinke A Müller HH Schade-Brittinger C Klose KJ Barth P Wied M Mayer C Aminossadati B Pape UF Bläker M Harder J Arnold C Gress T Arnold R PROMID Study Group Placebo-controlled, double-blind, prospective, randomized study on the effect of octreotide LAR in the control of tumor growth in patients with metastatic neuroendocrine midgut tumors: a report from the PROMID Study Group J Clin Oncol 2009 1;27 28 4656 4663 10.1200/JCO.2009.22.8510 19704057

[B19] Garcia AA Hirte H Fleming G Yang D Tsao-Wei DD Roman L Groshen S Swenson S Markland F Gandara D Scudder S Morgan R Chen H Lenz HJ Oza AM Phase II clinical trial of bevacizumab and low-dose metronomic oral cyclophosphamide in recurrent ovarian cancer: A trial of the California, Chicago, and Princess Margaret Hospital phase II consortia J Clin Oncol 2008 26 76 82 10.1200/JCO.2007.12.1939 18165643

[B20] Dellapasqua S Bertolini F Bagnardi V Campagnoli E Scarano E Torrisi R Shaked Y Mancuso P Goldhirsch A Rocca A Pietri E Colleoni M Metronomic cyclophosphamide and capecitabine combined with Bevacizumab in advanced breast cancer J Clin Oncol 2008 26 4899 4905 10.1200/JCO.2008.17.4789 18794539

[B21] Kulke MH Siu LL Tepper JE Fisher G Jaffe D Haller DG Ellis LM Benedetti JK Bergsland EK Hobday TJ Van Cutsem E Pingpank J Oberg K Cohen SJ Posner MC Yao JC Future directions in the treatment of neuroendocrine tumors: consensus report of the National Cancer Institute Neuroendocrine Tumor clinical trials planning meeting J Clin Oncol 2011 1;29 7 934 943 10.1200/JCO.2010.33.2056 PMC3068065 21263089

[B22] Rindi G Kloppel G Alhman H Caplin M Couvelard A de Herder WW Erikssson B Falchetti A Falconi M Komminoth P Körner M Lopes JM McNicol AM Nilsson O Perren A Scarpa A Scoazec JY Wiedenmann B and all other Frascati Consensus Conference participants European Neuroendocrine Tumor Society (ENETS) TNM staging of foregut (neuro)endocrine tumors: a consensus proposal including a grading system Virchows Arch 2006 449 4 395 401 10.1007/s00428-006-0250-1 16967267 PMC1888719

[B23] Simon R Optimal two-stage designs for phase II clinical trials Control Clin Trials 1989 10 1 10 10.1016/0197-2456(89)90015-9 2702835

[B24] Izzedine H Massard C Spano JP Goldwasser F Khayat D Soria JC VEGF signalling inhibition-induced proteinuria: mechanisms, significance and management Eur J Cancer 2010 46 2 439 448 10.1016/j.ejca.2009.11.001 20006922

[B25] de Boer IH Ioannou GN Kestenbaum B Brunzell JD Weiss NS 25-Hydroxyvitamin D levels and albuminuria in the Third National Health and Nutrition Examination Survey (NHANES III) Am J Kidney Dis 2007 50 69 77 10.1053/j.ajkd.2007.04.015 17591526

[B26] Buttigliero C Monagheddu C Petroni P Saini A Dogliotti L Ciccone G Berruti A Prognostic role of vitamin D status and efficacy of vitamin D supplementation in cancer patients: a systematic review Oncologist 2011 16 9 1215 1227 10.1634/theoncologist.2011-0098 21835895 PMC3228169

[B27] Bodei L Pepe G Paganelli G Peptide receptor radionuclide therapy (PRRT) of neuroendocrine tumors with somatostatin analogues Eur Rev Med Pharmacol Sci 2010 14 4 347 351 20496546

